# Effect of *Lactobacillus delbrueckii* subsp.* lactis* on vaginal radiotherapy for gynecological cancer

**DOI:** 10.1038/s41598-023-37241-7

**Published:** 2023-06-21

**Authors:** Zhichao Bi, Qi Wang, Tiancizhuo Yang, Yinhui Liu, Jieli Yuan, Longjie Li, Yanjie Guo

**Affiliations:** 1 Department of Microecology, School of Basic Medical Science, Dalian Medical University, Dalian, China; 2 Department of Radiation Oncology, The First Affiliated Hospital of Dalian Medical University, Dalian, Liaoning China

**Keywords:** Cancer, Microbiology, Medical research, Oncology

## Abstract

The aim of this study was to evaluate the effect of *Lactobacillus delbrueckii *subsp. *lactis (L.del)* on vaginal microbiota (VM) dysbiosis and vaginal radiation injury in gynecologic cancer patients. The inhibitory effects of *L.del* on cervical cancer cells were also studied in vitro. Gynecologic cancer patients receiving radiotherapy were randomized into control and *L.del* intervention groups. The control group received radiotherapy, while the intervention group received radiotherapy and *L.del* intervention (1 capsule/day placed into the deep vagina from the first day of radiotherapy until the end of treatment). Vaginal swab samples were collected on the first day pre-treatment and the last day post-treatment. DNA from 54 patients was extracted and assessed by the 16S rRNA sequencing method. Radiotherapy resulted in vaginal microbiome dysbiosis characterized by increased phylogenetic diversity and increased abundance of *Brevundimonas*, *Streptococcus* and *Prevotella*, but a decreased abundance of *Lactobacillus*. Level 2 vaginal radiation injury was positively associated with the abundance of *Brevundimonas* and gram-negative non-fermenting bacteria. Administration of *L.del* attenuated the reduction of *Lactobacillus* while also inhibiting the abundance of *Streptococcus* and *Prevotella*, thereby ameliorating radiotherapy-related vaginal microbiota dysbiosis. CLD inhibited the in vitro proliferation of SiHa cells by altering the expression of BCL2, HPV16-E6, HPV16-E7, IL6, MAP7, BAX, Caspase-3, Caspase-9 and LTF. In conclusion, *L. del* application can alleviate radiation-induced vaginal dysbiosis and restore *Lactobacillus* dominance of the vaginal microbiome. Moreover, CLD was found to inhibit cell growth and promote the apoptosis of SiHa cells in vitro. The registration number for this clinical trial is ChiCTR1900021784.

## Introduction

The increasing number of cervical cancer (CC) and endometrial cancer (EC) cases and the high mortality from these diseases are a serious concern for women's health. Worldwide, there were an estimated 604,127 new cases and 341,831 deaths attributed to CC in 2020, and a further 417,367 new cases and 97,370 deaths attributed to EC^1^. For the past few decades, radiotherapy has been the major common therapeutic regimen used in clinical practice for CC and EC. However, this treatment can lead to different degrees of radiation injury to patients.

Radiotherapy is associated with adverse reactions such as diarrhea, rectal bleeding, urinary tract bleeding, and vaginal radiation toxicity^2–4^. The effects of acute vaginal radiation toxicity manifest in the mucosa underlying the lamina propria and in small blood vessels. Common clinical manifestations include inflammation, dryness, hyperemia, swelling and increased vaginal discharge^5^. Late radiation toxicity effects include loss of capillaries, decreased endothelial cell proliferation and increased collagen production. These effects can develop months to years after radiotherapy and can manifest as mucosal atrophy, bleeding, vaginal shortening and/or narrowing, and increased susceptibility to injury by trauma or further radiation exposure^5,6^. These adverse vaginal sequelae may negatively affect the patient’s quality of life. Studies on treatments to reduce toxic side effects are lacking.

Recently, accumulating evidence has highlighted the influence of mutualistic bacterial communities, such as intestinal microbiota and vaginal microbiota, on human health^7,8^. The vagina is colonized by a diverse array of commensal microorganisms, of which *Lactobacillus* is the most abundant^9^. *Lactobacillus *spp. flourish in the anaerobic environment of the vagina, where epithelial cells metabolize glycogen to produce lactic acid, thereby keeping the vagina in a weak acidic environment. *Lactobacillus* play a protective role in the female reproductive tract by producing various antimicrobial compounds such as bacteriocin and hydrogen peroxide^10^. Disruption of the vaginal ecosystem is characterized by increased diversity of vaginal bacteria and decreased abundance of *Lactobacillus*. This is frequently associated with gynecological diseases^11^.

Gynecological tumor patients have distinct vaginal microbiota profiles compared to healthy women. Radiotherapy leads to even greater changes in the vaginal microbiota, characterized by the enrichment of opportunistic bacterial pathogens^12^. Radiation injures healthy mucosal cells and reduces estrogen-induced glycogen secretion, which is an important carbon source for *Lactobacillus*^13,14^. In addition, radiation leads to the release of necrotic material and to the recruitment of neutrophils, macrophages and mast cells^15–17^. These changes worsen the vaginal micro-environment, thus presenting challenges for the survival of vaginal microbiota. Since *Lactobacillus* is the most abundant bacteria in the vagina, we hypothesized that it may be more sensitive to changes in the micro-environment and show decreased abundance after radiotherapy. Following removal of a major competitor (*Lactobacillus*), other anaerobic bacteria gain the opportunity to thrive on the mucosal surface. Furthermore, it has been reported that individual bacterial taxa are strongly associated with reported vaginal symptoms. Low abundance of *Lactobacillus *sp. is associated with increased severity of vaginal dryness, while *Prevotella intermedia* is strongly associated with vaginal dryness. *Delftia *sp. is associated with high discomfort or pain following vaginal intercourse^18^. Therefore, maintaining the balance of vaginal microbiota is very important for the mitigation of radiation injury.

Several studies have found that probiotics combined with radiotherapy could inhibit microbiota dysbiosis and local inflammation of mucosal surfaces, such as the intestinal tract and oral cavity, by restoring micro-environmental homeostasis and regulating the immune system^19,20^. The use of probiotics to alleviate radiation-induced mucosal injury after vaginal radiotherapy has yet to be evaluated. *Lactobacillus delbrueckii *subsp. *lactis (L.del)* isolated from the vagina of healthy reproductive women is currently the only commercial preparation of *Lactobacillus* available. Previous studies revealed that *L.del* produced large amounts of H_2_O_2_ and inhibited *C. albicans* more effectively than many other *Lactobacillus* strains^21^.

In the present study, *L.del* was applied in gynecologic cancer patients receiving radiotherapy. The aim was to determine whether probiotics with *L.del* can reduce vaginal microbiota dysbiosis. In addition, we explored the relationship between changes in vaginal microbiota and vaginal radiotoxicity. We also studied the effect of *L.del* on tumor cells in vitro. This study may provide evidence to support the future use of probiotics in reducing vaginal radiation injury, thereby improving patient quality of life.

## Results

### Patient clinical characteristics

A total of 54 patients with gynecologic tumors were included in this study. Demographic characteristics and routine gynecological examination findings of the participants are summarized in Table 1. The control and intervention groups were similar in terms of age and tumor stage, as well as the prevalence of genital tract infections such as human immunodeficiency virus, candida vaginosis, trichomonas and bacterial vaginitis. There was no significant difference between the two groups in terms of the radiation dose (external beam radiotherapy dose and vaginal brachytherapy dose) during treatment, or in the vaginal swab pH, cleanliness and radiation injury level after treatment.

**Table 1:** Patients’ clinical characteristics.

	Control group (n = 26)	*L.del* intervention group (n = 28)	P value
Age (years, mean ± sd)	55.9 (± 9.9)	55.0 (± 7.8)	0.727
With tumor (n, %)	12 (46.2%)	7 (25.0%)	0.110
FIGO stage^a^ (n, %)			0.836
I	8 (30.8%)	9 (34.6%)	
II	11 (42.3%)	7 (26.9%)	
III	7 (26.9%)	10 (38.5%)	
Others	0 (0.0%)	2 (7.7%)	
Candida vaginosis (n, %)	0	0	–
Trichomonas (n, %)	0	0	–
Bacterial vaginitis (n, %)	0	0	–
External beam radiotherapy dose (Gy, mean ± sd)	47.2 (± 4.6)	46.7 (± 5.1)	0.709
Vaginal brachytherapy dose (Gy, mean ± sd)	19.0 (± 9.7)	15.9 (± 8.5)	0.187
Total radiation dose (Gy, mean ± sd)	66.2 (± 12.7)	62.6 (± 11.9)	0.286
Radiation injury level (post-treatment)	1.23 (± 0.43)	1.11 (± 0.31)	0.237
△Vaginal secretions pH (mean ± sem)	0.008 (± 0.057)	0.029 (± 0.075)	0.826
△Vaginal cleanliness (mean ± sem)	0.000 (± 0.124)	− 0.036 (± 0.209)	0.886

^a^Include cervical stump cancer and poor differentiated metrocarcinoma; △ Changes post-treatment compared with pre-treatment (post treatment -pre-treatment); *t*-test was used for statistical analysis.

### *L.del* application alleviated vaginal microbiota dysbiosis caused by radiation

**Figure 1:**
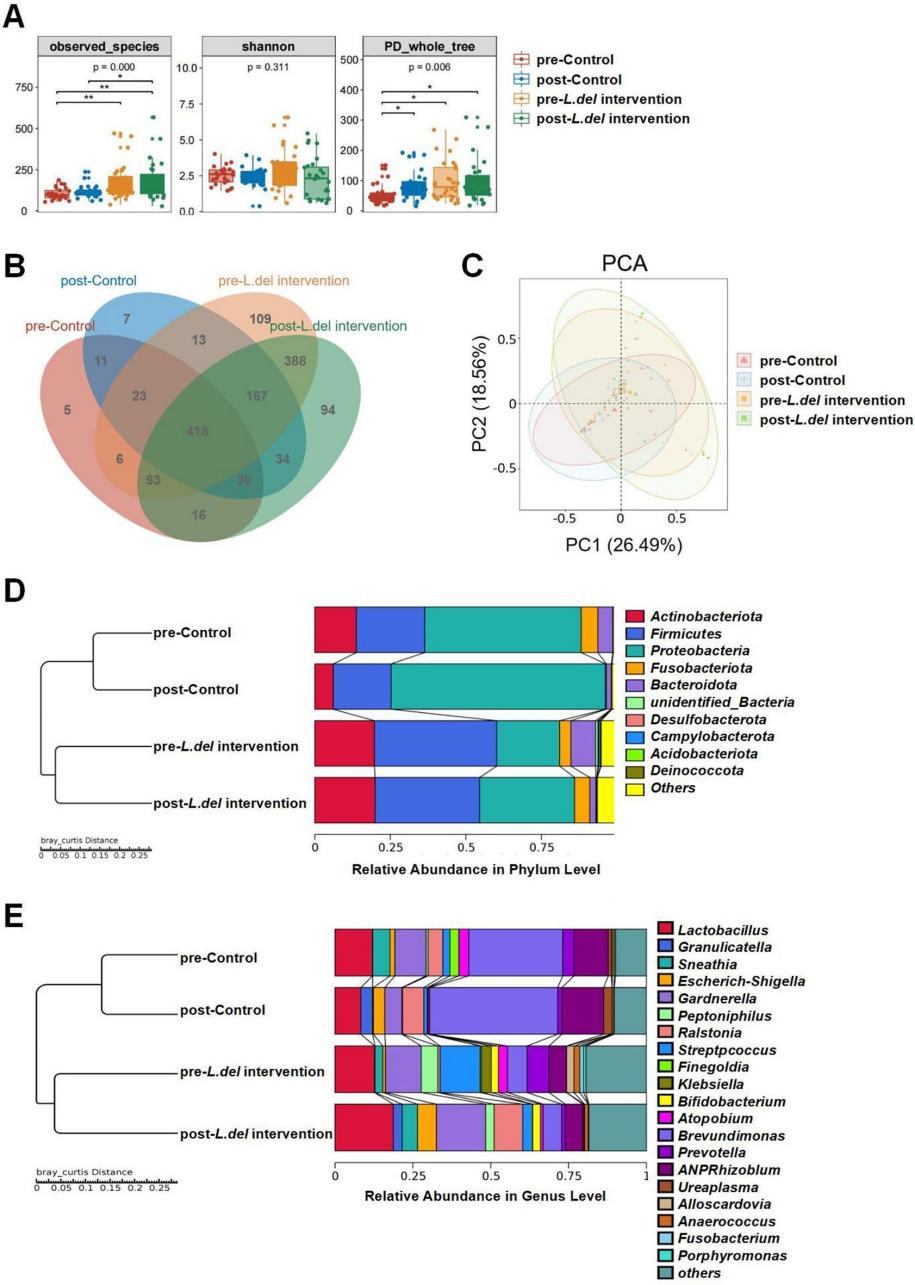
The effects of radiotherapy and *L.del* intervention treatment on vaginal microbiota composition and biodiversity. (**A**) The observed species number, Shannon diversity index and PD_whole_tree index of vaginal microbiota in different patient groups. The Alpha diversity indexes were calculated using Qiime software (V1.9.1, http://qiime.org/scripts/split_libraries_fastq.html)) and analyzed using R software (V2.15.3, http://www.R-project.org). Kruskal–Wallis test followed by *Dunn's* test was used for statistical analysis. *P < 0.05, **P < 0.01. (**B**) A scalar Venn representation of the vaginal microbiota. (**C**) Principal component analysis (PCA) using the weighted UniFrac distance metric in vaginal microbiota. Comparison of vaginal bacteria at the phylum level (**D**) and at the genus level (**E**). *Allorhizobium-Neorhizobium-Pararhizobium-Rhizobium* is shortened to *ANPRhizobium.*

All DNA samples from vaginal swabs were sequenced by 16S-rDNA in order to explore the impact of radiotherapy on vaginal microbiota and the therapeutic effects of *L.del* application. In the same patient group, the number of observed species and the Shannon index of α-diversity did not change significantly following radiotherapy. The phylogenetic diversity (PD) whole tree reflecting the richness and kinship of species increased significantly after radiotherapy in the control group, but not in the *L.del* intervention group (Fig. 1A).

Changes in OTUs before and after treatment were analyzed in each group by Venn diagram (Fig. 1B). There were 602, 703, 1217 and 1274 OTUs in the pre-control, post-control, pre-*L.del* intervention and post-*L.del* intervention groups, respectively. These four groups shared a total OTU abundance of 418, occupying 69.4%, 59.5%, 34.3% and 33.7% in each group, respectively. Weighted Unifrac PCA of all samples at the genus levels is shown in Fig. 1C. Inter-individual differences in the vaginal microbiota were found to exceed the treatment effect, and both treatments caused the point distribution to deviate from the direction of the second to the fourth quadrant. To identify the most altered microbes resulting from vaginal radiotherapy and *L.del* intervention, we compared the abundance of various bacterial groups at the phylum and genus levels. The main dominant phyla identified in all samples were Proteobacteria, Firmicutes, Actinobacteria, Fusobacteria and Bacteroidetes. Vaginal radiotherapy led to an increase in Proteobacteria and a decrease in Bacteroidetes in all patients. In the control group, radiotherapy decreased Actinobacteria (p = 0.014), whereas the changes in the *L.del* intervention group were not significant (Fig. 1D and supplementary Tables S2 and S3). At the genus level, the relative abundance of *Prevotella,* decreased and *Escherichia coli-Shigella* increased in all patients. In the control group, vaginal brachytherapy decreased the abundance of *Lactobacillus* and increased opportunistic *Brevundimonas* (p = 0.019)*.* Conversely, *L.del* intervention increased the abundance of *Lactobacillus* and decreased the abundance of *Streptococcus* (p = 0.0402) (Fig. 1E and supplementary Tables S2 and S3).

**Figure 2:**
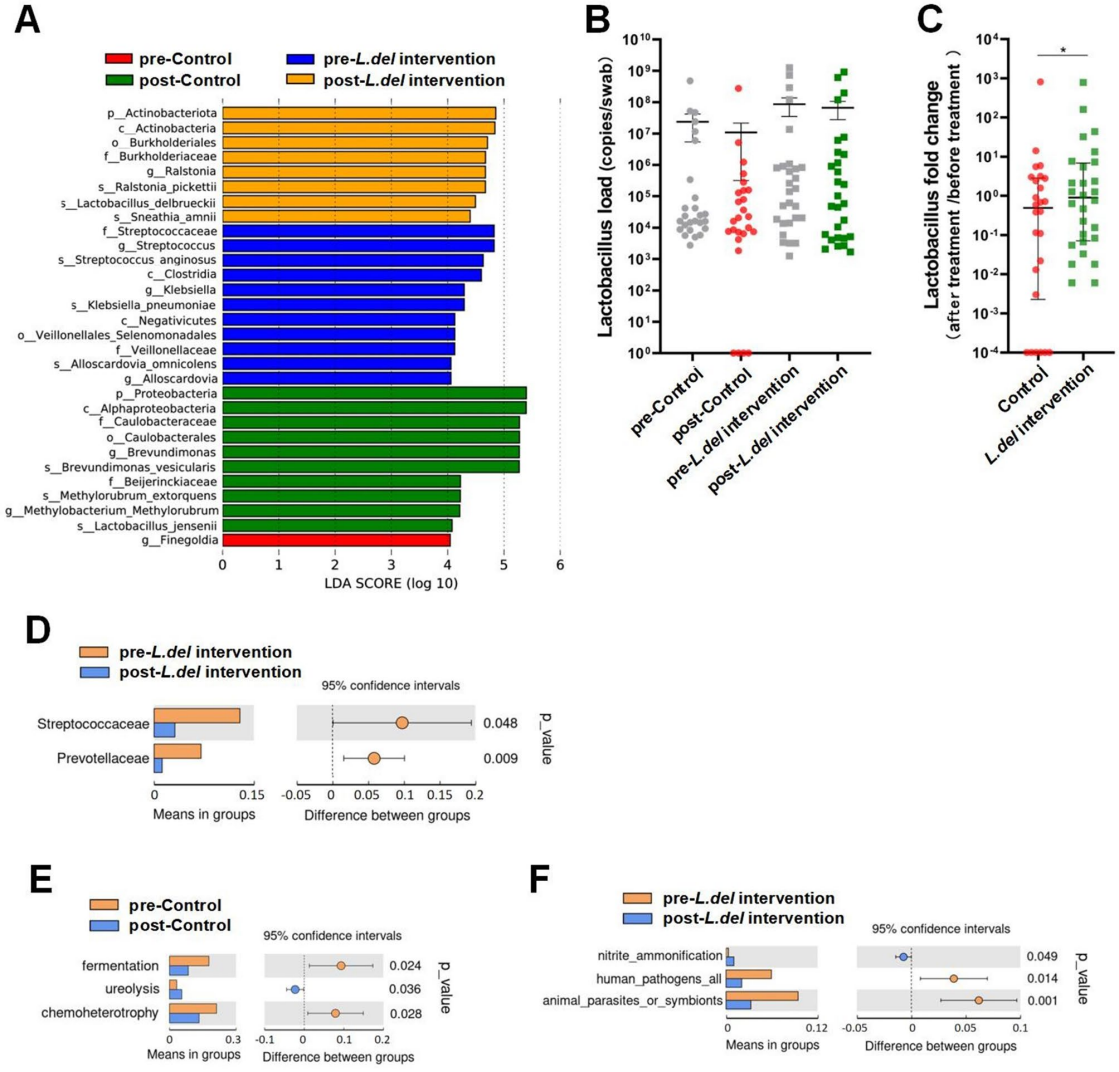
Differences in bacteria and function between patient groups. (**A**) The linear discriminant analysis effect size (LefSe) was used to identify bacterial groups that showed significant differences in abundance among the four groups. (**B**) The *Lactobacillus* load in all samples was tested by absolute quantitative RT-PCR. Data are presented as the mean ± SEM. (**C**) The change in *Lactobacillus* load in all patients pre- and post- treatment was evaluated by absolute quantitative RT-PCR. The median and IQR are shown. *P < 0.05. (**D**) T-test of different bacteria between groups. (**E**,**F**) TAX4FUN functional prediction for vaginal microbiota in the control (**E**) or *L.del* intervention (**F**) groups, and T test of the difference between pre- and post- treatment vaginal microbiota.

The linear discriminant analysis effect size (LefSe) was applied to further evaluate vaginal microbiota abundance differences between assigned taxa^22^. The results showed that *Brevundimonas vesicularis* of *Proteobacteria* had the strongest association with radiotherapy. *L.del* intervention was associated with changes in the vaginal microbiota from *Streptococcus* and *Klebsiella* to *Lactobacillus delbrueckii* and *Ralstonia* (Fig. 2A).

In view of the large differences in total amount of bacteria from vaginal swabs of different patients, relative abundance changes may not fully reflect the impact of treatment on the absolute amount of *Lactobacillus* in the vagina. The whole genus level *Lactobacillus* load in all patients was examined by absolute q-PCR, with the post-/pre-treatment ratio then used to assess the change in whole *Lactobacillus* load in each patient. A slight decrease in *Lactobacillus* load was observed in both groups after radiotherapy (Fig. 2B). However, the *Lactobacillus* load post-/pre-treatment ratio in the control group decreased significantly compared to the *L.del* intervention group (Fig. 2C). TAX4FUN was then used to predict the function of vaginal microbiota^23^, with the results showing that radiation decreased bacteria with fermentation and chemo-heterotrophy functions in the control group, and increased bacteria with ureolysis functions. Moreover, the number of potential pathogens and parasite functional profiles decreased significantly after treatment in the *L.del* intervention group (Fig. 2D–F).

### Radiation-induced vaginal mucosal injury was associated with vaginal microbiota CST-C

**Figure 3:**
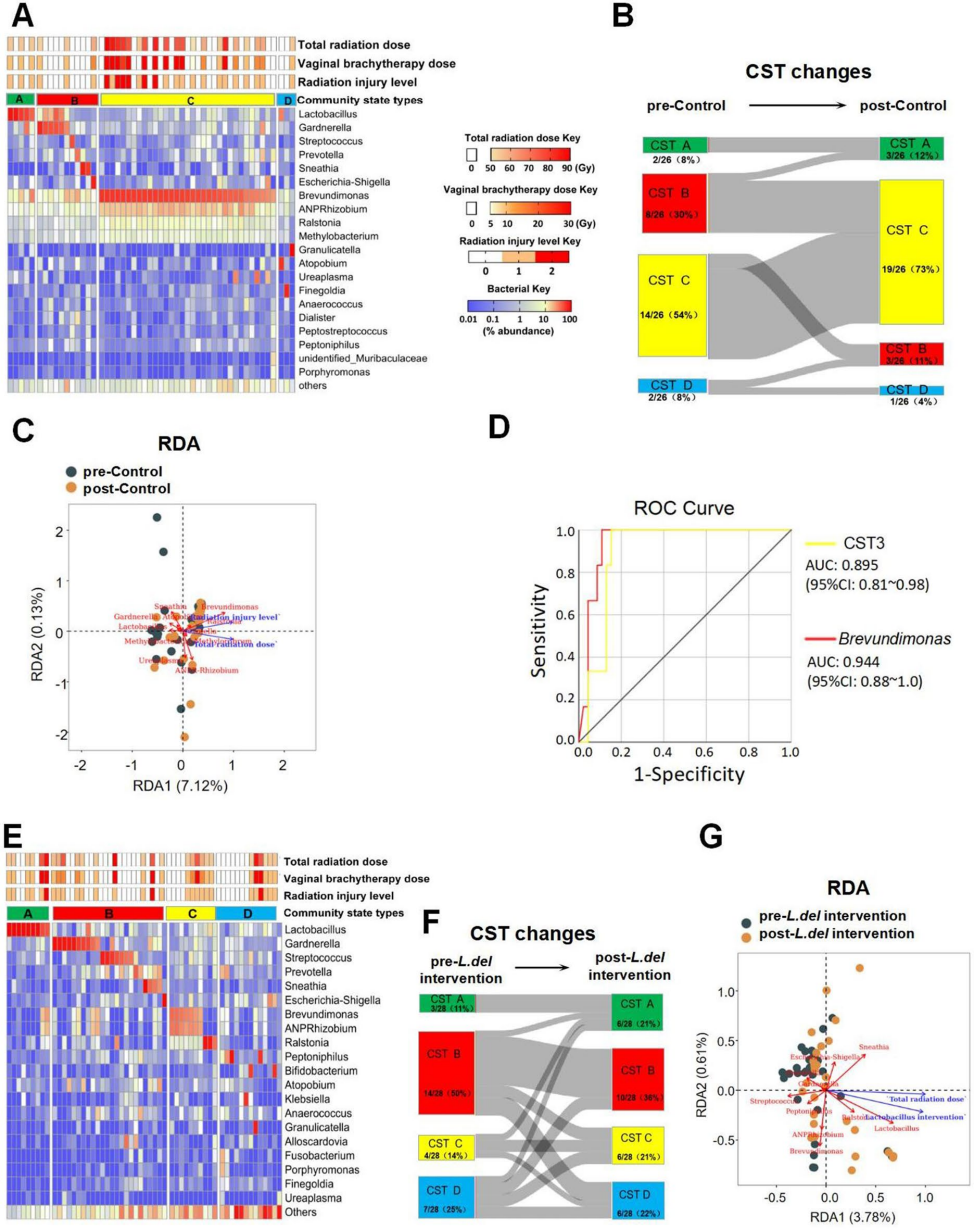
Relationship between vaginal microbiota composition and clinical indexes. (**A**) Heatmaps of bacterial taxa, total radiation dose, vaginal brachytherapy dose and radiation injury level in the control group are shown. (**B**) Changes in the CST before and after radiotherapy in the control group. (**C**) The relationship between vaginal microbiota, total radiation dose, and radiation injury level in the control group was analyzed by redundancy analysis (RDA). Each dot in the figure represents a sample, the blue-arrow represents the clinical characteristics, and the red-arrow represents the genus level of bacteria. The length of the arrow represents the magnitude of the impact on vaginal microbiota, the angle between the two arrows represents their correlation (0–180° linear corresponding R = 1 to − 1). (**D**) Receiver operating characteristic (ROC) curves for evaluating the ability to predict radiation injury level 2 using CST-C dominant and *Brevundimonas* abundance in the control group. Each curve represents the sensitivity and specificity for distinguishing radiation injury level 2 from radiation injury level 1. (**E**) Heatmaps of bacterial taxa, total radiation dose, vaginal brachytherapy dose, and radiation injury level are shown for the *L.del* intervention group. (**F**) Pre- and post-radiotherapy CST changes in the *L.del* intervention group. (**G**) RDA of the relationship between vaginal microbiota, total radiation dose, and *Lactobacillus* intervention in the *L.del* intervention group.

The vaginal microbiota CST of all patients was analyzed to evaluate the relationship between vaginal microbiota and clinical indices. In the pre-treatment control group, the incidence of CST-A, -B, -C and -D was 7.7% (2/26), 30.8% (8/26), 53.8% (14/26), and 7.7% (2/26), respectively. After radiotherapy, all patients showed varying degrees of vaginal injury. Six patients (dominated by *Brevundimonas*) belonging to CST-C presented with level 2 radiation vaginal injury. *ANPRhizobium*, *Ralstonia* and *Methylobacterium* appeared to be always accompanied by *Brevundimonas* (Fig. 3A). Following treatment, the incidence of CST-A, -B, -C and -D was 11.5% (3/26), 11.5% (3/26), 73.1% (19/26), and 3.8% (1/26), respectively. The Sankey diagram was used to visualize changes in CST after treatment. This is a specific type of flow chart in which the width of the extended branch corresponds to the volume of patient flow^24^. The results showed that patients who were CST-A prior to radiotherapy remained CST-A after radiotherapy. Seven of the 8 patients who were CST-B changed to CST-C after radiotherapy, making CST-C the most common CST after radiotherapy (Fig. 3B).

To further investigate the correlation between radiation dose, vaginal microbiota and radiation injury degree, redundancy analysis (RDA) was performed on samples from control patients after radiotherapy (Fig. 3C). This revealed the degree of radiation injury was significantly and positively correlated with radiation dose (R > 0.6, P < 0.05). Furthermore, increased radiation dose was accompanied by a reduced abundance of *Lactobacillus*, *Gardnerella*, *Sneathia* and *Prevotella*, and increased abundance of the ‘CST-C dominant bacteria’ *Brevundimonas*, *Anprhizobium*, *Ralstonia* and *Methylobacterium* (P < 0.05). Receiver operating characteristic (ROC) curve analysis showed that CST-C and *Brevundimonas* could distinguish radiation injury level 2 from radiation injury level 1, with an AUC of 0.895 (95% CI 0.808–0.982, p < 0.01) and 0.944 (95% CI 0.882–1, p < 0.01), respectively (Fig. 3D).

### Application of *L.del* can prevent the transformation of the vaginal microbiota to CST-C induced by radiotherapy.

The pre-treatment incidence of CST-A, -B, -C and -D in the *L.del* intervention group was 10.7% (3/28), 50% (14/28), 14.3% (4/28), and 25% (7/28), respectively. Three patients were diagnosed with radiation injury level 2 after receiving combined treatment (Fig. 3E). The incidence of CST-A, -B, -C and -D after treatment was 21.4% (6/28), 35.7% (10/28), 21.4% (6/28), and 21.4% (6/28), respectively. The Sankey diagram showed that patients who received combined treatment did not have a clear target for CST changes (Fig. 3F). Only 3 patients changed to CST-C and these switched to *Ralstonia*-dominated CST-C rather than *Brevundimonas*-dominated CST-C. Furthermore, RDA results showed that *L.del* treatment was positively correlated with abundance of *Lactobacillus, Bifidobacterium* and *Sneathia*, whereas abundance of *Brevundimonas, Anprhizobium, Ralstonia* and *Prevotella* were negatively correlated with *L.del* treatment (Fig. 3G).

### *L.del* application has a better effect on vaginal microbiota adjustment in patients receiving radical radiotherapy compared to those receiving adjuvant radiotherapy

To better understand the effects of *L.del* application on vaginal microbiota dysbiosis, we compared pre/post treatment changes in the vaginal microbiota of patients treated with adjuvant radiotherapy and radical radiotherapy in both the control and *L.del* intervention groups. Patients were divided into four groups according to the radiotherapy regimen (adjuvant radiotherapy or radical radiotherapy): A-control (control group patients who received adjuvant radiotherapy), R-control (control group patients who received radical radiotherapy), A-*L.del* intervention (*L.del* intervention group patients who received adjuvant radiotherapy), and R-*L.del* intervention (*L.del* intervention group patients who received radical radiotherapy). The clinical characteristics of patients in each of these four groups are shown in Table 2.

**Table 2:** Characteristics of adjuvant radiotherapy and radical radiotherapy patients.

	Control group			*Lactobacillus* intervention group		
	A-Control (n = 14)	R-Control (n = 12)	P value	A-*Lactobacillus* intervention (n = 21)	R-*Lactobacillus* intervention (n = 7)	P value
Age (years, mean ± sd)	52.4 (± 10.0)	59.9 (± 8.3)	0.048	54.1 (± 8.0)	57.8 (± 7.1)	0.295
Radiation dose (Gy, mean ± sd)	55.8 (± 3.1)	78.3 (± 7.6)	0.001	56.6 (± 4.8)	80.6 (± 7.1)	0.001
Radiation injury level (after treatment, mean ± sd)	1.00 (± 0)	1.50 (± 0.52)	0.001	1.00 (± 0)	1.43 (± 0.53)	0.001

A-, patients who received adjuvant radiotherapy; R-, patients who received radical radiotherapy; *t*-test was used for statistical analysis.

**Figure 4:**
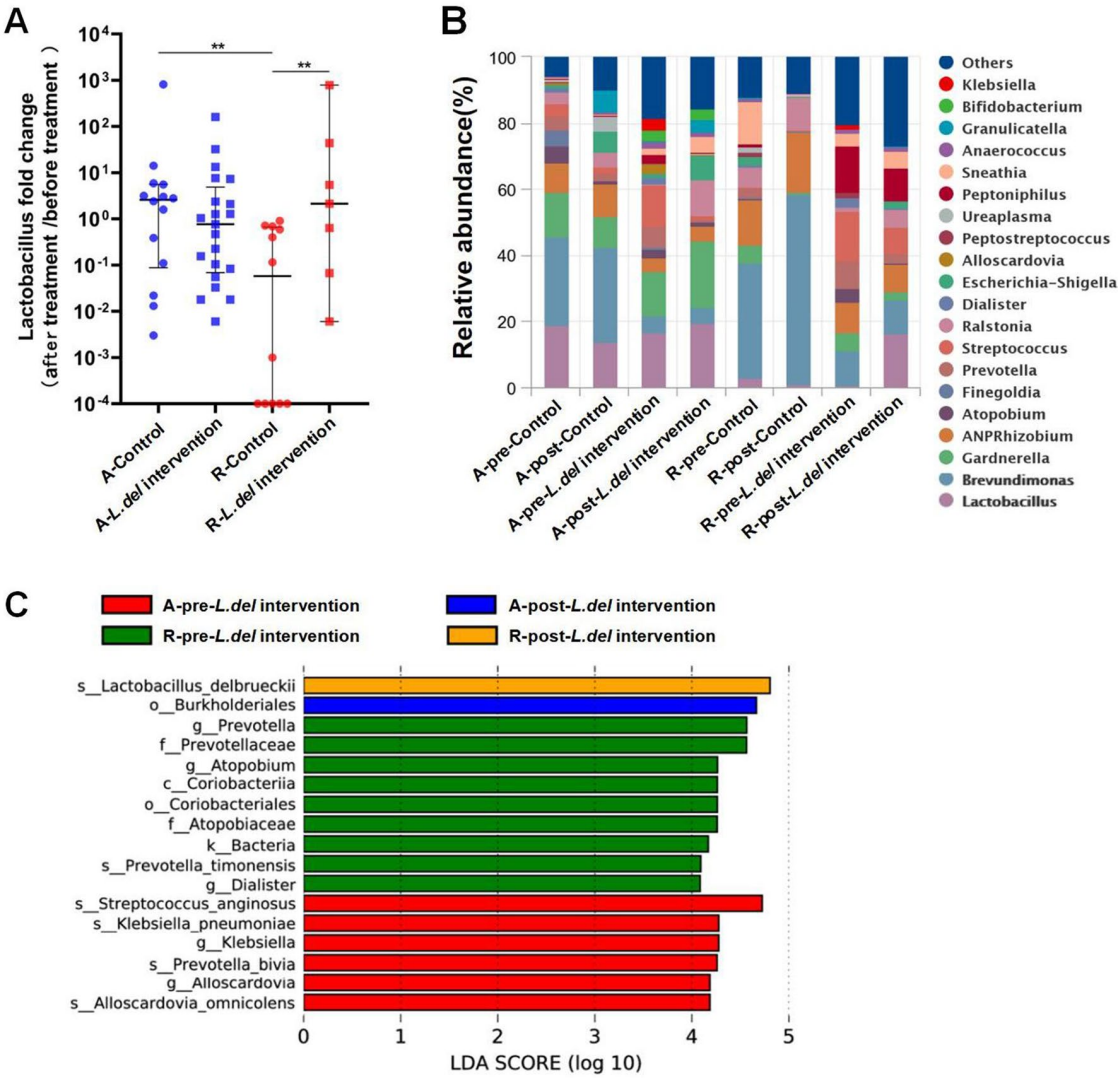
Vaginal microbiota analysis. (**A**) Pre- and post-treatment *Lactobacillus* load changes in different groups. The median and IQR are shown. T-test was used for statistical analysis. **P < 0.01. (**B**) Relative abundance of bacteria at the genus level in vaginal microbiota. (**C**) Histograms of LDA scores were used to identify bacteria that differed significantly between groups.

The whole genus level *Lactobacillus* load post-/pre-treatment ratio revealed that patients who received radical radiotherapy had a more significant reduction in *Lactobacillus* load after radiotherapy compared to patients who received adjuvant radiotherapy (A-control group vs R-control group, P < 0.01). *L.del* intervention could not prevent the reduction of *Lactobacillus* load in patients who received adjuvant radiotherapy (A-control group vs A-*L.del* intervention group). However, *L.del* intervention significantly reversed the reduction of *Lactobacillus* in patients who received radical radiotherapy (R-control group vs R-*L.del* intervention group) (Fig. 4A).

The results of vaginal microbiota analysis at the genus level showed that the average abundance of *Lactobacillus* in patients who received radical radiotherapy was as low as 5% prior to treatment. Radical radiotherapy further reduced the abundance of *Lactobacillus* and increased the proportion of *Brevundimonas*. However, *L.del* intervention significantly increased the abundance of *Lactobacillus* and decreased *Brevundimonas*. In addition, a difference in the pathogen clearance effect of *L.del* intervention was observed between patients who received the two radiotherapy regimens. *L.del* intervention resulted in stronger inhibitory effects on *Gardnerella* and *Sneathia* in R-*L.del* patients compared to A-*L.del* patients, but was less effective in inhibiting *Streptococcus* (Fig. 4B). LefSe analysis confirmed that the abundance of *Lactobacillus delbrueckii* increased more significantly in R-*L.del* patients. In addition, *L.del* intervention markedly reduced *Streptococcus* in patients who received adjuvant radiotherapy, and reduced *Prevotella* in patients who received radical radiotherapy (Fig. 4C).

### *L.del* cytoplasmic (CLD) inhibited cell proliferation and promoted apoptosis of SiHa cell in vitro

**Figure 5:**
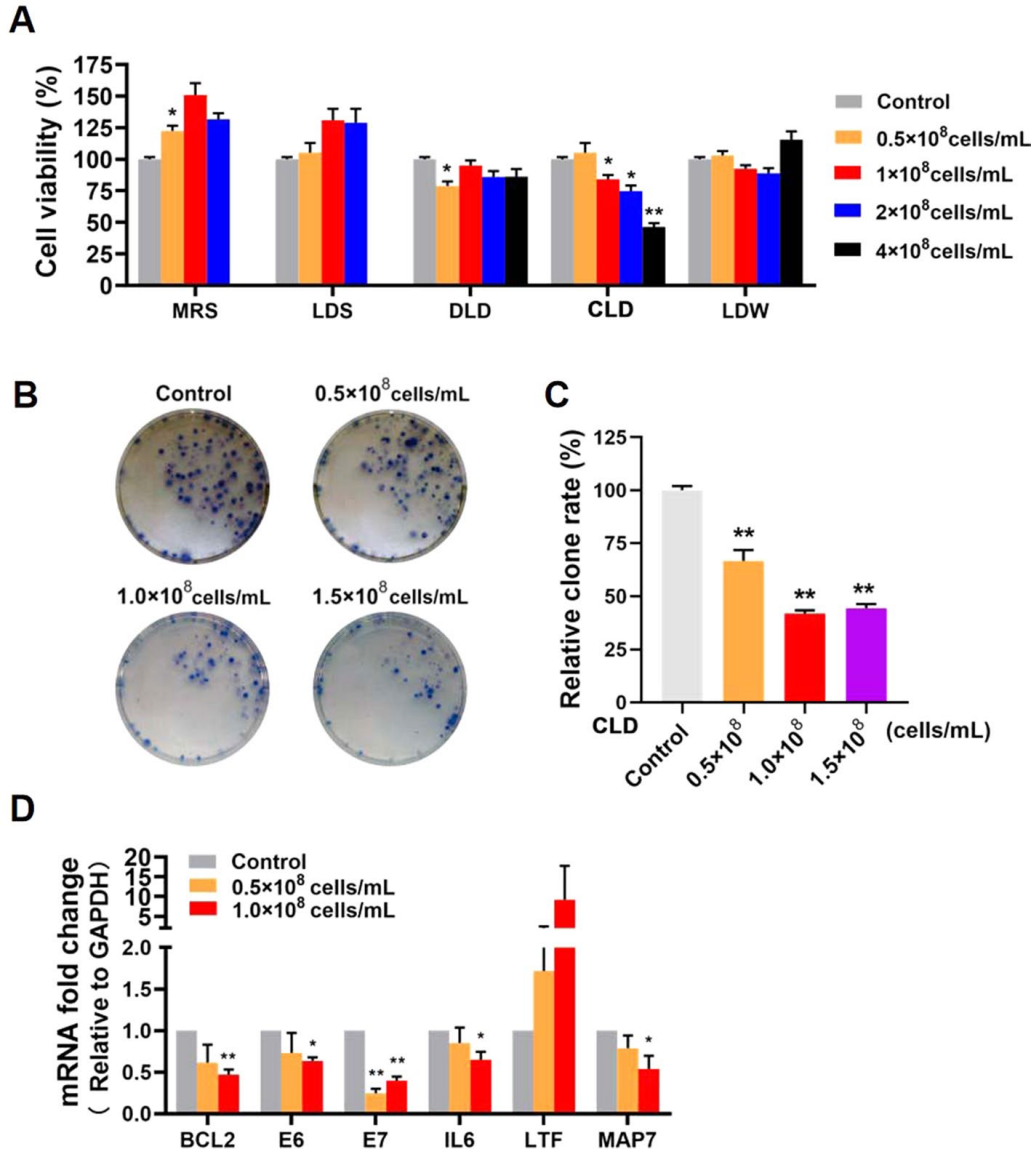
CLD promoted the proliferation of SiHa cells in vitro. (**A**) Effects of *L.del* components and supernatant on the proliferation of cervical cancer cells in vitro. Data are shown as the mean ± SEM and are compared with the control group. *P < 0.05, **P < 0.01. (**B**,**C**) Colony formation assays. (**D**) Fold-changes in the expression of mRNA for cancer progression-related genes in SiHa cells treated with CLD for 48 h. One way ANOVA was used for statistical analysis.

Vaginal administration of *L.del* can result in direct contact with cervical cancer tissue. The effects of *L.del* on the growth of cervical cancer cells in vitro were examined by MTT assay and colony formation assay. CLD was found to suppress SiHa cell growth in a concentration dependent manner (Fig. 5A–C). mRNA levels for the cancer progression-related genes HPV16-E6, HPV16-E7, IL6, and MAP7 were significantly downregulated in SiHa cells treated with CLD compared to control cells, while LTF was upregulated (Fig. 5D).

**Figure 6:**
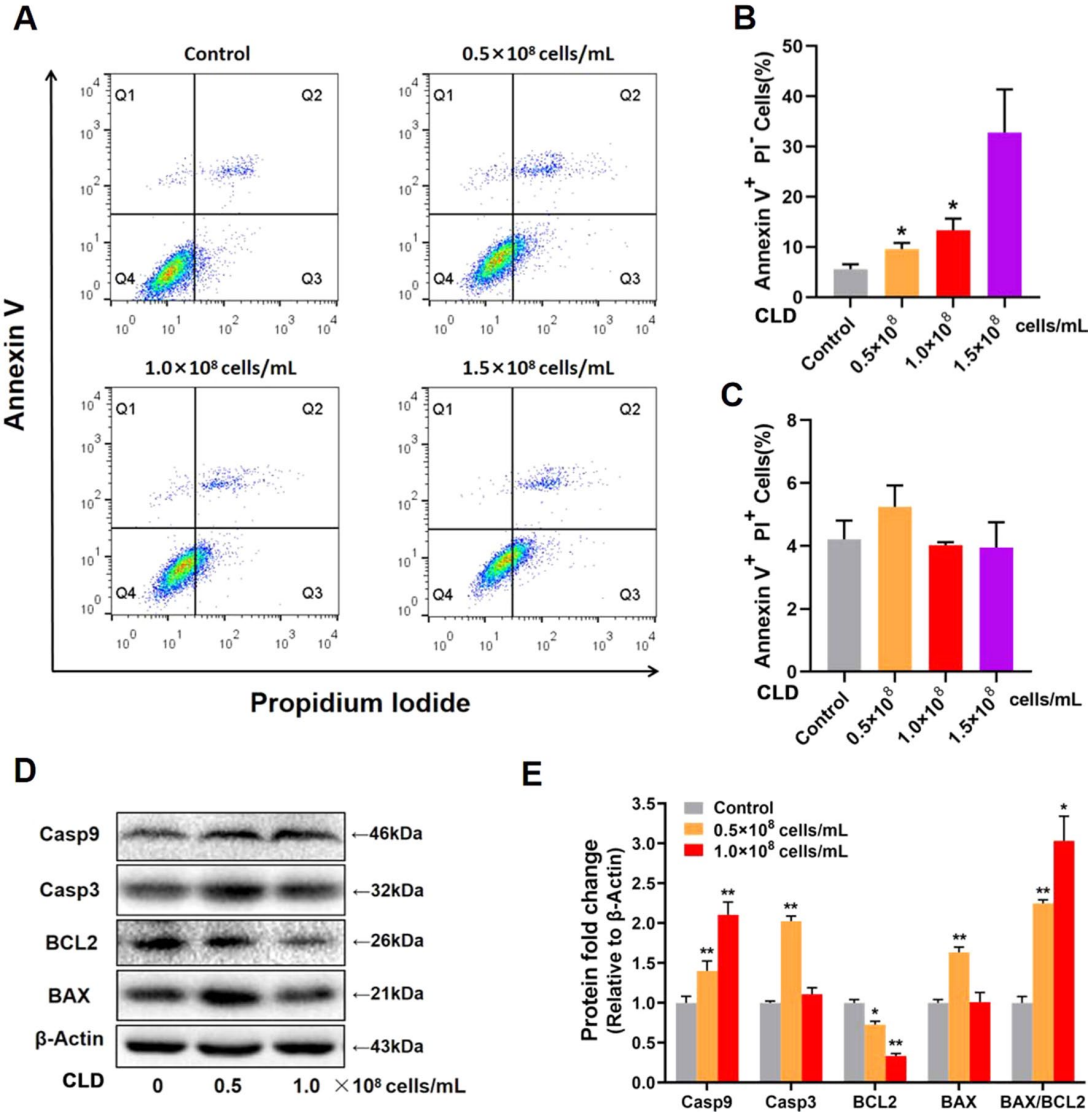
CLD promoted the apoptosis of SiHa cells in vitro. (**A**) Cell apoptosis was detected by flow cytometry. (**B**,**C**) Percentage of late apoptotic cells. Data are shown as the mean ± SEM and are compared with the control group *P < 0.05, **P < 0.01. (**D**,**E**) Expression of apoptosis-related proteins in SiHa cells treated with CLD for 48 h. The original blots are shown in Fig. S1. One way ANOVA was used for statistical analysis.

Since E6, E7, MAP7 and LTF are also related to tumor cell apoptosis^25–27^, we used flow cytometry to further evaluate SiHa cell apoptosis after treatment with CLD. Compared to the control group, CLD treatment significantly increased the number of early apoptotic cells in a dose-dependent manner (Fig. 6A–C). Expression of the apoptosis pathway-related genes Caspase-9, Caspase-3, BAX and BAX/BCL2 index was significantly increased in CLD-treated SiHa cells (Fig. 6D,E). These results indicate that CLD could have anti-tumor effects by inhibiting cell proliferation and promoting apoptosis.

## Discussion

*Lactobacillus* plays an important role in maintaining female reproductive health. Reproductive diseases such as vaginitis, HPV infection and cervical cancer are often associated with increased vaginal microbiota diversity and decreased abundance of *Lactobacillus*^11,28,29^. The novel therapeutic strategy of targeting vaginal microbiota dysbiosis through vaginal microbiota transplantation or probiotics intervention has shown some benefits for the treatment of female genital tract disorders^11,30^. In this context, we hypothesized that adjuvant *L.del* intervention may reduce damage to the vaginal microenvironment caused by radiotherapy.

Radiotherapy was found to result in vaginal microbiota dysbiosis, as observed by increased phylogenetic diversity (PD), decreased *Lactobacillus* abundance, and an increased abundance of opportunistic pathogens represented by *Brevundimonas*. *L.del* application partly restored vaginal microbiota dysbiosis, as observed by a decline in *Lactobacillus*, reduced proliferation of *Brevundimonas,* and reduced abundance of the *Streptococcus* and *Prevotella* pathogens. Reconstitution of damaged vaginal microecology following *L.del* application was also reported in patients with bacterial vaginosis (BV)^31^. The restoration effect of *L.del* on vaginal microecology may be due to its strong ability to produce various anti-infection agents, including lactic acid and hydrogen peroxide.

In conjunction with host cells, vaginal microbiota can produce unique fermentation metabolic by-products. Metabolic products such as lactic acid, acetate, butyrate, and propionate can influence host susceptibility to genital tract disorders such as bacterial vaginosis, vulvovaginal candidiasis and aerobic vaginitis^16,32^. In the present study, data on the prediction of bacterial community function showed that radiotherapy decreased the fermenter functional profiles and increased the urea-decomposing functional profiles. Furthermore, the abundance of pathogenic functional profiles was significantly reduced in the *L.del* intervention group. The reduction of fermentation enzymes may reduce the production of lactic acid and short-chain fatty acids, while increased urease has been shown to aggravate intestinal mucosal inflammation^33^. Changes in the structure and function of vaginal microbiota may therefore underlie radiotherapy-induced vaginal damage and account for the beneficial effects of probiotic *L.del* intervention.

We also investigated the association between vaginal microbiota changes, radiation dose, and radiation injury level. CST was used to classify vaginal microbiota with different characteristics. CST-C dominated by *Brevundimonas*, *ANPRhizobium* and *Ralstonia* was the most common pre-radiotherapy CST for patients in the control group. Radiotherapy partially converted some CST-B to CST-C, while further increasing the amount of CST-C. *Brevundimonas* spp. can form resilient sessile biofilms and are thought to be opportunistic pathogens. Previous studies have reported that some *Brevundimonas* spp. cause serious infections in individuals with underlying medical conditions^34,35^. *Ralstonia* has also been reported as an opportunistic bacterium found in the water environment, with increasing incidence as a nosocomial pathogen^36,37^. However, a definitive role for *Ralstonia* is still unclear, with some studies reporting an association with chronic endometritis, HPV infection and cervical intra-epithelial neoplasia (CIN). *Ralstoni*a has also been correlated with a decreased risk of early preterm birth^38,39^. In the current study, the patient’s vagina was very dry and the load of microbes was low after radiotherapy. In this case, there may be an invasion of opportunistic pathogens or the CST-C may due to environmental microbial contamination. The limitation of this study is that no negative control swabs were sequenced to remove environmental contamination. In this study, RDA correlation analysis showed that *Brevundimonas* was significantly and positively associated with high radiation dose and radiation injury level 2. CST-C, and especially a high-abundance of *Brevundimonas*, could thus be useful as a predictor of radiation injury level 2.

After combined treatment with *Lactobacillus* in the *L.del* intervention group, the vaginal microbiota of most patients retained the initial CST instead of changing to CST-C. In line with the change of CST, the incidence of radiation injury level 2 in the *L.del* intervention group was lower than in the control group (10.7% [3/28] vs 23.1% [6/26]). Importantly, three patients with different initial CSTs were converted to CST-A after combination therapy. The enhanced ureolysis function after radiotherapy can be explained by the increase in gram-negative bacteria with urease function (*Brevundimonas*, *ANPRhizobium* and *Ralstonia*)^40,41^. The conversion of urea to ammonia and carbon dioxide catalyzed by bacterial urease is an important cause of gastric mucositis, intestinal mucositis and increased oral pH^33,42,43^. This suggests that CST-C induced by radiotherapy may produce inflammatory and toxic effects on the vaginal mucosa. The therapeutic effects of *L.del* intervention were attenuation of the reduction in *Lactobacillus*, alteration of the vaginal microbiota to CST-C, and reduction of radiation injury level 2 caused by radiotherapy. Moreover, *L.del* intervention promoted *Lactobacillus* as the dominant vaginal microbiota in some patients.

In the present study cohort, 64.8% (35/54) of patients received adjuvant radiotherapy and 35.2% (19/54) received radical radiotherapy. Radical treatment uses a higher radiation dose, thereby resulting in greater radiotoxicity compared to adjuvant treatment. *L.del* intervention significantly increased the relative abundance of *Lactobacillus* and decreased the relative abundance of *Streptococcus* and *Prevotella* in the vaginal microbiota of patients who received radical radiotherapy, while also reducing the incidence of radiation injury level 2. However, there were no significant beneficial effects of *L.del* intervention in patients who received adjuvant radiotherapy. These data suggest that the more severe the damage to the vaginal ecology caused by radiotherapy, the better the recovery effect of probiotics with *L.del*. The underlying mechanism for this benefit is still unclear, but may be related to colonization resistance.

*Lactobacillus* dominance is associated with vaginal health, and the depletion of these microorganisms can lead to various diseases including gynecological cancer. Modulation of the microbiome using probiotics may improve the responsiveness to cancer treatment^44^. In addition to exploring the effect of *Lactobacillus* on the vaginal microbiota of patients in the current study, the effect on cervical cancer cell growth was also studied in vitro. Our results showed that CLD could inhibit cell proliferation and promote cell apoptosis in SiHa cells by downregulating the expression of HPV16-E6, HPV16-E7, IL6, and MAP7, and by upregulating BAX, Caspase-3, Caspase-9 and LTF. It has been previously demonstrated that E6 and E7 are the most important oncogenes for high-risk HPV. LTF could act as a tumor suppressor by inhibiting the AKT signaling pathway or by releasing BAX^27,45^. MAP7 belongs to the family of microtubule-associated proteins (MAPs). Its depletion in cervical cancer cells can increase the expression of Caspase 3 and BAX, while reducing that of BCL2^25,46^. These gene expression changes suggest that CLD may affect the mitochondrial apoptosis pathway of tumor cells, which is one of the two main apoptosis mechanisms that have been reported. Mitochondrial apoptosis is caused by release into the cytosol of the contents of the mitochondrial intermembrane space, including cytochrome C and SAMC. BAX and BAK oligomers participate in the release of these contents, with BCL2 sabotaging the pore-forming activity of BAX. Therefore, the ratio of BAX/BCL2 is crucial to the outcome of apoptosis^47,48^. In the present study, CLD significantly increased the BAX/BCL2 ratio in SiHa cells and activated Caspase-9, which may in turn lead to activation of Caspase-3 in the cytoplasm, thus inducing apoptosis.

In conclusion, our work demonstrated that radiation therapy for patients with gynecological cancer resulted in vaginal microbiome dysbiosis characterized by a decrease in *Lactobacillus* and an increase in gram-negative, non-fermenting bacteria. Moreover, the degree of vaginal mucosa injury was related to changes in the vaginal microbiota. Secondly, adjuvant therapy with *L.del* could attenuate the vaginal microbiome disorder caused by radiation therapy and restore the dominance of *Lactobacillus* in the vagina. Better therapeutic effects with *L.del* were observed in patients with radical radiotherapy. Finally, *L.del* cytoplasmic extract can inhibit cell growth and promote apoptosis in SiHa cells. Further studies using *Lactobacillus* in large patient cohorts treated with radical radiation therapy are required to confirm these observations.

## Materials and methods

### Patient enrollment and study design

Eighty women with pathologically-diagnosed gynecological cancer were enrolled in this study. Written consent was obtained from all patients to provide information and samples for research purposes. The inclusion criteria were: (1) female patients aged 18–85 years; (2) pathological diagnosis of cervical or endometrial cancer; (3) ECOG score > 2 and an estimated survival time of > 3 months; (4) Dalian local Chinese Han population ethnicity. The exclusion criteria were: (1) diagnosed with suppurative and chronic infection, or non-healing wound; (2) diagnosed with disease of the immune system (chronic nephritis, rheumatism, rheumatoid diseases, allergic eczema, HIV infection, or other disease); (3) abnormal coagulation function or other blood system diseases; (4) pregnancy, lactating, or fertile but not taking effective contraceptive measures; (5) antibiotics or probiotics used within the previous two weeks.

**Figure 7:**
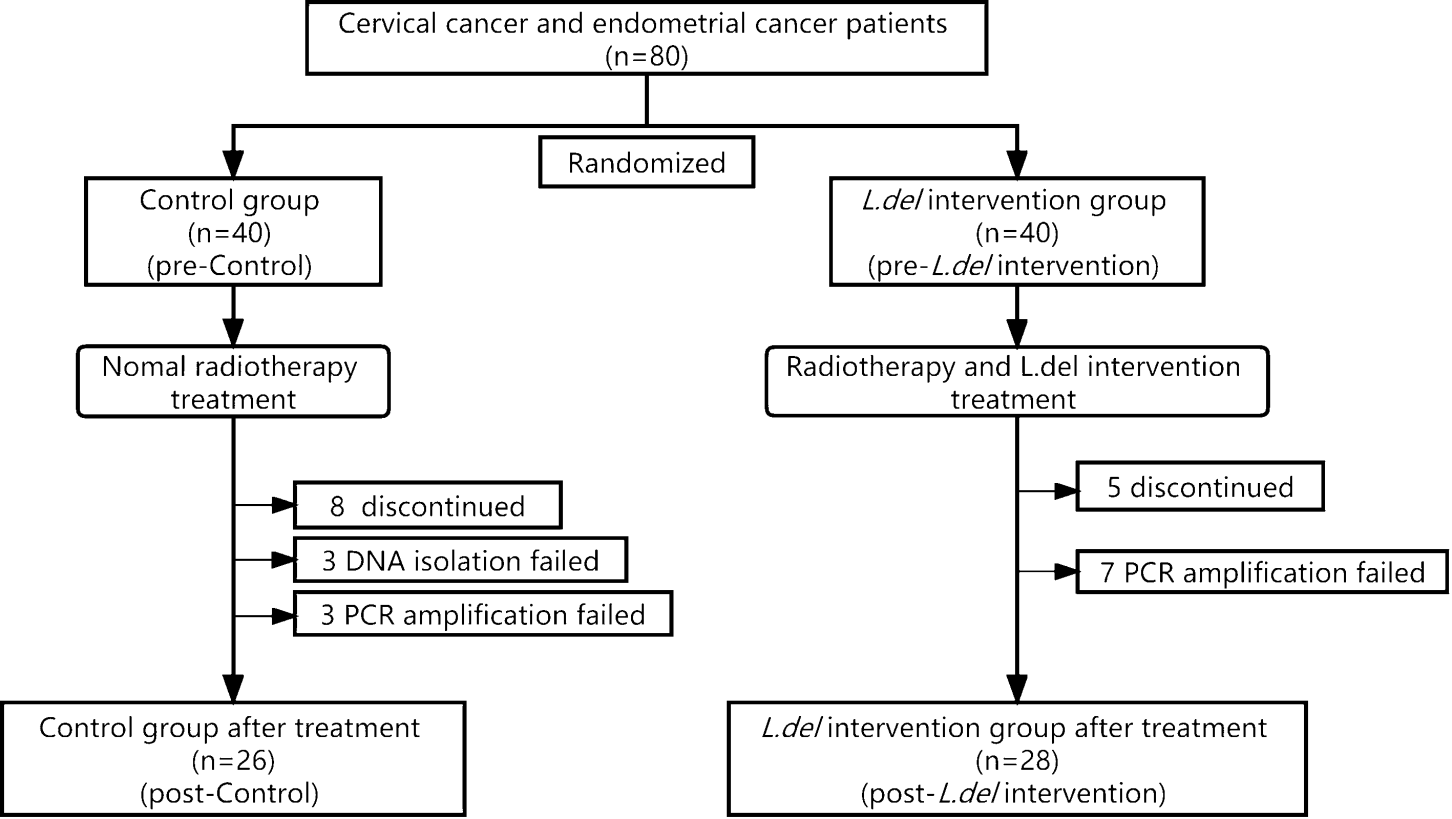
Study design.

Participants were randomly divided into the control group and the *L.del* intervention group, as shown in Fig. 7. Patients were scheduled for pelvic external beam radiotherapy (EBRT) and brachytherapy. EBRT was performed in a linear accelerator AXESSE (ELEKTA), using 6 MV energy. The EBRT dose received by patients was 45 Gy, with a daily fraction of 1.8 Gy. For grossly positive nodal disease, this was boosted up to 60.2 Gy (2.15 Gy/fraction). Intracavitary brachytherapy (IBT) was delivered in 3–5 fractions and given twice weekly. The time to completion of radiotherapy was 42–60 days. From the first day of radiotherapy until the last day of radiotherapy, patients in the *L.del* intervention group were treated with *Lactobacillus* (Live *Lactobacillus* Capsule for Vaginal Use; Inner Mongolia Shuangqi Pharmaceutical Co. Ltd, China) according to the manufacturer’s instruction (capsule placed deep into the vagina, 1 capsule/day, *L.del* > 2.5 × 10^5^ CFU/capsule). Eight patients in the control group and five in the *L.del* intervention group discontinued the study. DNA isolation failed in 3 patients from the control group due to a dry vagina following radiation therapy. DNA amplification failed in 3 patients from the control group and 7 patients from the *L.del* intervention group. Finally, 26 patients were included in the control group and 28 in the *L.del* intervention group.

### Sample collection and clinical characteristics

Lateral wall vaginal swab samples were collected on the first day pre-treatment and the last day post-treatment. Two swabs were collected each time, with one being sent to the laboratory for routine gynecological examination using a leucorrhea analyzer (BD, Franklin Lakes, New Jersey) and a bacterial vaginosis test kit (Boyang Biotechnology Co., Ltd, China). The other swab was placed in a sterile empty cryovial and stored at -80℃ until testing. While collecting the vaginal swabs, the radiation injury of the vaginal mucosa was graded by a designated gynecologist according to Radiation Therapy Oncology Group (RTOG) standards^49^.

### DNA extraction and 16S rDNA sequencing

Vaginal swab DNA was extracted using the QIAamp DNA Mini Kit (QIAGEN, Hilden, Germany) and amplified by PCR using the 16S V4 region primer 515F (5′ GTGCCAGCMGCCGCGGTAA 3′), 806R (5′ GGACTACHVGGGTWTCT AAT 3′). The PCR product was purified using GeneJET gel extraction assay (Thermo Scientific). TruSeq^®^ DNA PCR-Free Sample Preparation Kit was used to generate the sequencing library. The library was quantified using Qubit Assay (Life Technologies) and Q-PCR. The concentration of a qualified library should be > 2 nM without adapter sequences. Barcoded amplicons were sequenced by the paired-end method using NovaSeq6000 (Illumina, San Diego, California). After sequencing, Barcode and sequencing primers were trimmed from the amplicons. Raw reads were obtained using FLASH (V1.2.7, http://ccb.jhu.edu/software/FLASH/) to splice the sequence. The assembled sequence was quality-filtered using Qiime (V1.9.1, http://qiime.org/scripts/split_libraries_fastq.html). Sequences with three continuous fuzzy bases, or with lengths < 150 bp were removed. Clean reads were obtained after removing the chimera sequences using UCHIME Algorithm (http://www.drive5.com/usearch/manual/uchime_algo.html) and Gold database (http://drive5.com/uchime/uchime_download.html).

### Taxonomy classification and statistical analysis

The clean reads were clustered into Operational Taxonomic Units (OTUs) at 97% similarity using the Uparse algorithm (Uparse v7.0.1001, http://www.drive5.com/uparse/). The OTUs were classified to species using the Mothur method and the SSUrRNA database (http://www.arb-silva.de/), with a threshold of 0.8–1. Taking into account the difference in sequencing volume of different samples, the data were corrected for sequencing depth by randomly resampling all samples in the OTU abundance matrix according to the lowest sequencing depth. This was done to obtain a Rarefied OTU abundance matrix at the same sequencing depth. The Alpha diversity indexes Observed_species, Shannon and PD_whole_tree were calculated using Qiime software (Version 1.9.1) and analyzed using R software **(**V2.15.3**,** http://www.R-project.org**)**. Beta diversity was calculated using the weighted UniFrac distance. Principal component analysis (PCA) based on the UniFrac distance was performed using R software. The linear discriminant analysis (LDA) effect size (LefSe) was performed to identify microbial taxa that differed significantly between the two groups, with use of a threshold LDA score of > 4. TAX4FUN^23^ was used to predict functional profiles of the microbial community based on the prokaryotic KEGG Orthology reference profile and the SILVA databas^50–52^.

The vaginal microbiota composition of healthy, reproductive-age women was classified initially into four vaginal community state types (CSTs) I–IV based on the dominant bacterial species at genus level according to previous studies^9,53,54^. In the present study, the patients were perimenopausal and postmenopausal women with cervical or endometrial cancer. The structure of the vaginal microbiome was quite different from healthy reproductive-age women. The *Lactobacillus* dominated community occurred in only 13% (14/108) of the total vaginal swabs. Therefore, in the present study, the vaginal bacterial communities were grouped according to the patients’ vaginal community composition. The top 20 bacterial genera were used for analysis. The bacteria with the highest abundance in each sample were defined as the dominant bacteria. We identified four distinct vaginal community types amongst all of the patients in this study. CST-A were dominated by *Lactobacillus*, CST-B by inflammation-related pathogens including *Gardnerella*, *Streptococcus*, *Prevotella*, *Sneathia* and *Escherichia-Shigella*, CST-C by non-fermentative gram-negative bacilli including *Brevundimonas*, *Allorhizobium-neorhizobium-pararhizobium-rhizobium* (*ANPRhizobium*) and *Ralstonia*, and CST-D by other bacteria or mixed bacteria. Redundancy analysis (RDA) was carried out to explore correlations between species distribution and clinical indices.

### Absolute quantitative analysis of *Lactobacillus*

Real-time PCR was used for the absolute quantification of *Lactobacillus*. As described previously^55^, standards were prepared from the gDNA of *L.del*. Each sample or standard was tested in triplicate in a 20 μL reaction system. The PCR reaction conditions and primer sequences are shown in the supplementary materials. Standard curves were generated using tenfold dilutions of *L.del* genomic DNA from 5.56 × 10^8^ copies/µL to 5.56 × 10^3^ copies/µL. The test data for each sample was substituted into the standard curve (r^2^ > 0.999) to obtain the genomic copy number data for 1 μL DNA extract, and then converted into the genome copy number for each swab (copies/swab). These data were analyzed using Mxpro software (Agilent, Santa Clara, California).

### *L.del* culture and isolation of components

*L.del* was isolated from *Lactobacillus* capsules and amplified in MRS broth medium (Solarbio, Beijing, China) under microaerobic conditions at 37 °C. To obtain *L.del* components, *L.del* was cultured for 12 h then centrifuged at 8000×*g* for 10 min. The supernatant was collected for use as *L.del* supernatant. The pellet was washed and resuspended in PBS at a concentration of 5 × 10^10^ cells/mL. The bacterial suspension was divided into two parts. One was inactivated at 121 °C for 15 min to obtain dead *L.del*. The other was disrupted by sonication with an Ultrasonic pulverizer (Bilon, Shanghai, China) on ice at 150 W for 8 s at 10 s intervals to obtain *L.del* homogenates. To obtain *L.del* cytoplasmic (CLD) extracts and *L.del* cell wall extracts, aliquots of the homogenates were ultracentrifuged at 27,000×*g* for 20 min at 4 °C. The resulting supernatant was the cytoplasm component and the pellet was the cell wall component. All bacterial components except for dead *L.del* were filtered through a 0.2 µm membrane and used for subsequent experiments.

### Cell culture and cell proliferation assays

The SiHa cell line (human cervical squamous carcinoma cell) was purchased from Zhongqiao Xinzhou Biotechnology (Shanghai, China) and cultured in DMEM hyperglycemic medium (Thermo Gibco, Waltham, Massachusetts) containing 10% fetal bovine serum (BI, Israel) and 100 U/mL penicillomycin (Solarbio, Beijing, China) in humid air at 37 °C and 5% CO_2_.

A total of 1 × 10^3^ cells/well were seeded into 96-well plates in triplicate. After 24 h, *L.del* components were added at concentrations of 0.5 × 10^8^ cells/mL, 1.0 × 10^8^ cells/mL, 2.0 × 10^8^ cells/mL and 4.0 × 10^8^ cells/mL. Cell proliferation was determined 48 h later using the MTT Kit (Seven Biotech, Beijing, China) according to the manufacturer’s instructions. Absorbance was measured using a Microplate photometer (Thermo, Waltham, Massachusetts).

### Colony formation assay

SiHa cells were seeded at 600 cells/well into 6-well plates and were then exposed to 0.5 × 10^8^ cells/mL, 1.0 × 10^8^ cells/mL and 1.5 × 10^8^ cells/mL CLD. After 48 h, all wells were replaced with fresh medium. Two weeks later, the colonies were immobilized with methanol and stained with Giemsa. Colonies with a diameter > 1.5 mm were counted.

### Real time RT-PCR

SiHa cells were exposed to 0.5 × 10^8^ cells/mL or 1.0 × 10^8^ cells/mL CLD for 48 h. Total RNA was extracted with Trizol RNA isolation reagents (TransGen Biotech; Beijing, China), followed by cDNA synthesis with a cDNA Reverse Transcription Kit (TransGen Biotech; Beijing, China). The qPCR was conducted with a 10 μL reaction system containing 10 ng of cDNA, 5 μL of SYBR qPCR Mix (TransGen Biotech; Beijing, China), 0.4 μL of primers (10 μM), 0.2 μL of ROX, and ddH_2_O. A Mx3005P qPCR instrument (Agilent; Santa Clara, California) was used for detection, and data were analyzed using Mxpro software (Agilent; Santa Clara, California). Gene expression levels were normalized to β-Actin. Relevant primer sequences and reaction conditions are shown in Table S1.

### Western blot analysis

SiHa cells were exposed to 0.5 × 10^8^ cells/mL or 1.0 × 10^8^ cells/mL of CLD for 48 h. Total protein was then extracted from the SiHa cells using RIPA Lysis buffer (Seven Biotech; Beijing, China) according to the manufacturer’s instructions and the protein concentration determined using the BCA protein assay kit (Seven Biotech; Beijing, China). Equal amounts of protein from each sample were separated by 10% sodium dodecyl sulfate polyacrylamide gel electrophoresis (SDS-PAGE) and transferred to a nitrocellulose membrane (Pall Corporation; NYC, New York). The membranes were incubated with antibodies for BAX, BCL-2, Caspase 9, or Caspase 3 (Protein tech, China) at 4 ℃ overnight, with antibody to β-Actin (Beyotime; China) used as the control. The optical density of specific bands was detected using an electrochemiluminescence detection system, and the relative protein expression level quantified by densitometry using Image Lab software (Bio-Rad, Berkeley, California).

### Cell apoptosis analysis

SiHa cells were exposed to 0.5 × 10^8^ cells/mL, 1.0 × 10^8^ cells/mL or 1.5 × 10^8^ cells/mL CLD for 48 h. An annexin V-FITC/PI apoptosis detection kit (Seven Biotech; Beijing, China) was used to evaluate both early and terminal apoptosis, as described by the manufacturer. Cells were stained with 5 μL annexin V-FITC and 5 μL PI in darkness for 30 min at room temperature. Immediately after Annexin-V/PI staining, samples were analyzed by FACS Calibur flow cytometry (BD, Franklin Lakes, New Jersey).

### Statistical analysis

Statistical analyses t-test, Wilcoxon test, Kruskal–Wallis test followed by *Dunn's* test and one-way ANOVA were performed using GraphPad Prism 8 Software (GraphPad, San Diego, California). *P* value less than 0.05 was considered statistically significant.

### Ethics statement

This study was conducted in accordance with the Declaration of Helsinki, and approved by the Ethics Committee of The First Affiliated Hospital of Dalian Medical University. This trial was registered on 09/03/2019 with the trial registration number ChiCTR1900021784.

## Supplementary Information

Supplementary Figures.

Supplementary Table S1.

Supplementary Table S2.

Supplementary Table S3.

## Data Availability

The datasets generated and analyzed during the current study are available in the [SRA] repository, (https://www.ncbi.nlm.nih.gov/sra/PRJNA881083).
