# Supplementary material for: Effect of Lactobacillus delbrueckii subsp. lactis on vaginal radiotherapy for gynecological cancer
Source: Sci Rep. 2023 Jun 21;13:10105. doi: 10.1038/s41598-023-37241-7 (PMC10284825; doi:10.1038/s41598-023-37241-7)
Supplement: Supplementary file 1 — Supplementary Figures. [file 41598_2023_37241_MOESM1_ESM.pdf]

Supplementary Figure 1

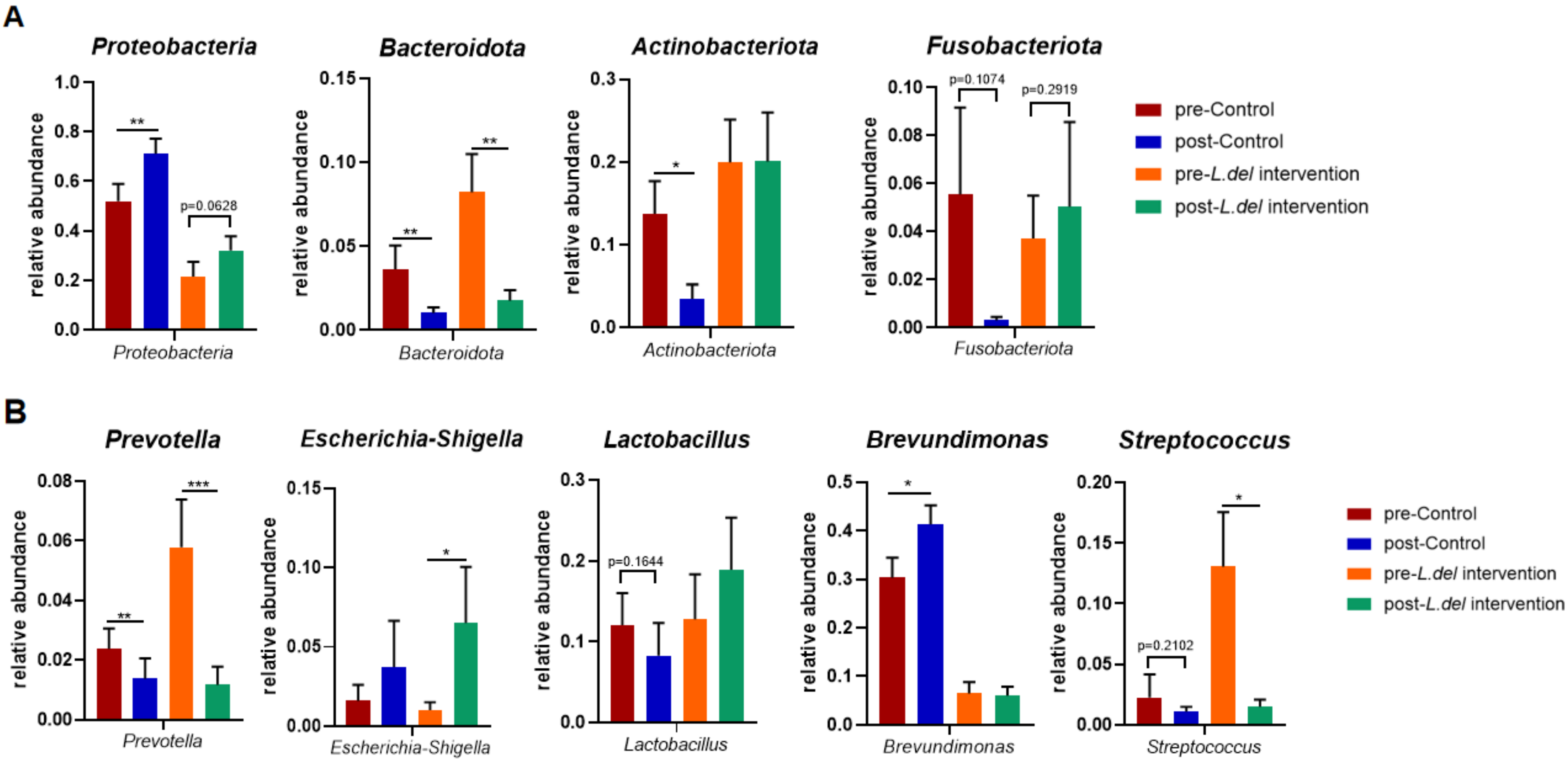

Supplementary Figure 2

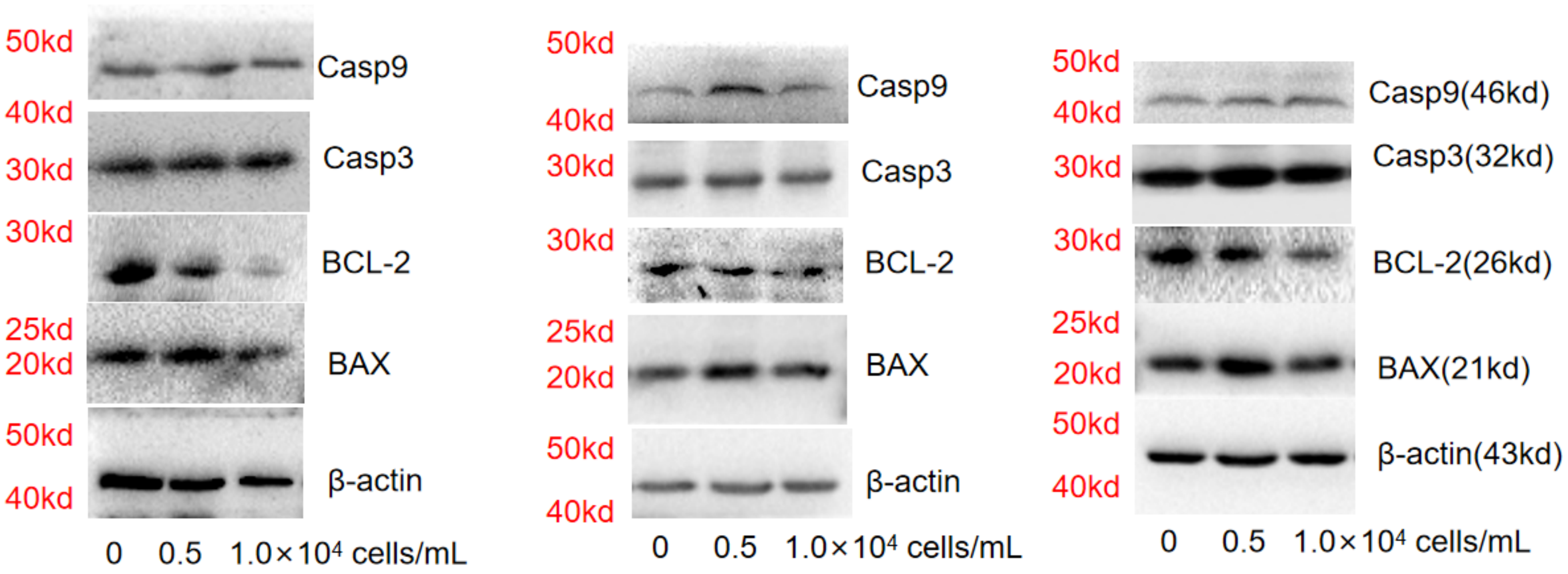

Note: In order to save antibodies, the blots were cut prior to hybridisation with antibodies. During western blotting experiment, protective proteins were added on both sides of the target protein to prevent the bands from skewing during electrophoresis. To avoid misleading the readers, we cut off the protective bands.
