# Supplementary material for: Effect of Lactobacillus delbrueckii subsp. lactis on vaginal radiotherapy for gynecological cancer
Source: Sci Rep. 2023 Jun 21;13:10105. doi: 10.1038/s41598-023-37241-7 (PMC10284825; doi:10.1038/s41598-023-37241-7)
Supplement: Supplementary file 2 — Supplementary Table S1. [file 41598_2023_37241_MOESM2_ESM.docx]

Table.S1 Primer information

| Primer name | Primer | Primer sequence (5'-3') | Product length (bp) | PCR Reaction conditions |
| --- | --- | --- | --- | --- |
| BAX | Forward | GGGACGAACTGGACAGTAACA | 297 | 95 ℃ 5 min +  (95 ℃ 15 s +  60 ℃ 1 min) × 40 cycles |
|  | Reverse | CCGCCACAAAGATGGTCAC |  |  |
| BCL2 | Forward | GGAGAGTGCTGAAGATTG | 116 |  |
|  | Reverse | ACTTCCTCTGTGATGTTGTA |  |  |
| Casp6 | Forward | CACCAACATAACTGAGGTGGATG | 170 |  |
|  | Reverse | AGGAGGAGCCATATTTTCCCA |  |  |
| Casp8 | Forward | GACTGGATTTGCTGATTACCTACCTAA | 143 |  |
|  | Reverse | CCTCAATTCTGATCTGCTCACTTCT |  |  |
| Casp9 | Forward | CCAGATGCCACCCCGTT | 184 |  |
|  | Reverse | CCCACTGCTCAAAGATGTCGT |  |  |
| GAPDH | Forward | CTGCACCACCAACTGCTTAG | 105 |  |
|  | Reverse | TTCTGGGTGGCAGTGATG |  |  |
| MAP7 | Forward | AAACTCTTTGTAACACCACCTGA | 134 |  |
|  | Reverse | GATGGAGATACAGCCCTTCG |  |  |
| NDRG1 | Forward | CTCCTGCAAGAGTTTGATGTCC | 127 |  |
|  | Reverse | TCATGCCGATGTCATGGTAGG |  |  |
| RBM38 | Forward | CTGCCGTACCACACTACCG | 176 |  |
|  | Reverse | ATGATGGGGTTCGGGTCTTTG |  |  |
| TRADD | Forward | CTTGCGCCATTTGAGACCC | 110 |  |
|  | Reverse | CGCCACCTGCCCAGACTTT |  |  |
| UEB2C | Forward | GACCTGAGGTATAAGCTCTCGC | 150 |  |
|  | Reverse | CAGGGCAGACCACTTTTCCTT |  |  |
| AIMP1 | Forward | AGTGGCCTGGTGAATCATGTT | 158 | 95 ℃ 5 min +  (95 ℃ 15 s +  55 ℃ 30 s +  72 ℃ 30 s) ×  40 cycles |
|  | Reverse | TTGTGAGGAGCCAAGATTTCAAT |  |  |
| BIN1 | Forward | CAAGTCCCCATCTCAGCCAG | 296 |  |
|  | Reverse | GGATCACCAGCACCACATCA |  |  |
| HPV16-E6 | Forward | GACCCAGAAAGTTACCACAG | 130 |  |
|  | Reverse | CATAAATCCCGAAAAGCAAAG |  |  |
| HPV16-E7 | Forward | GGAGGAGGATGAAATAGATGG | 189 |  |
|  | Reverse | TGAGAACAGATGGGGCACAC |  |  |
| IL6 | Forward | ACTCACCTCTTCAGAACGAATTG | 149 |  |
|  | Reverse | CCATCTTTGGAAGGTTCAGGTTG |  |  |
| LTF | Forward | ATGGTGGTTTCATATACGAGGCA | 79 |  |
|  | Reverse | CTTTCGGTCCCGTAGACTTCC |  |  |
| p53 | Forward | GCTTTCCACGACGGTGAC | 97 |  |
|  | Reverse | GCTCGACGCTAGGATCTGAC |  |  |
| PTEN | Forward | TGAACTGCTAGCCTCTGGATTTGA | 170 |  |
|  | Reverse | TAGAGCGTGCAGATAATGACAAGGA |  |  |
