# Supplementary material for: Effect of Lactobacillus delbrueckii subsp. lactis on vaginal radiotherapy for gynecological cancer
Source: Sci Rep. 2023 Jun 21;13:10105. doi: 10.1038/s41598-023-37241-7 (PMC10284825; doi:10.1038/s41598-023-37241-7)
Supplement: Supplementary file 3 — Supplementary Table S2. [file 41598_2023_37241_MOESM3_ESM.docx]

Table S2 Relative abundance of vaginal microbiota at phylum level

| Taxonomy | pre-Control | post-Control | pre-*L.del* intervention | | post-*L.del* intervention | pre-Control  vs  post-Control | pre-*L.del* intervention vs  post-*L.del* intervention |
| --- | --- | --- | --- | --- | --- | --- | --- |
| Phylum (relative%) |  |  |  | |  | *P* | *P* |
| *Proteobacteria* | 0.52±0.07 | 0.71±0.06 | 0.22±0.06 | 0.32±0.06 | | 0.026 | 0.206 |
| *Firmicutes* | 0.23±0.04 | 0.19±0.05 | 0.41±0.06 | 0.35±0.06 | | 0.4082 | 0.3272 |
| *Actinobacteria* | 0.14±0.04 | 0.06±0.03 | 0.2±0.05 | 0.2±0.06 | | 0.141 | 0.989 |
| *Fusobacteria* | 0.06±0.04 | 0.00±0.00 | 0.04±0.02 | 0.05±0.04 | | 0.223 | 0.740 |
| *Bacteroidetes* | 0.05±0.02 | 0.02±0.01 | 0.08±0.02 | 0.02±0.01 | | 0.085 | 0.085 |
| *Deinococcota* | 0.00±0.00 | 0.00±0.00 | 0.00±0.00 | 0.00±0.00 | | 0.6807 | 0.9915 |
| *Campylobacterota* | 0.00±0.00 | 0.00±0.00 | 0.01±0.00 | 0.00±0.00 | | 0.2938 | 0.372 |
| *Cyanobacteria* | 0.00±0.00 | 0.00±0.00 | 0.00±0.00 | 0.00±0.00 | | 0.3125 | 0.2630 |
| *unidentified_Bacteria* | 0.00±0.00 | 0.00±0.00 | 0.01±0.00 | 0.00±0.00 | | 0.0985 | 0.3271 |
| *Desulfobacterota* | 0.00±0.00 | 0.00±0.00 | 0.00±0.00 | 0.00±0.00 | | 0.0625 | 0.5219 |
| *Others* | 0.01±0.00 | 0.01±0.00 | 0.04±0.01 | 0.06±0.03 | | 0.0357 | 0.3502 |

Values are presented as means + SEM, Wilcoxon test was used for statistical analysis.
