# Supplementary material for: Effect of Lactobacillus delbrueckii subsp. lactis on vaginal radiotherapy for gynecological cancer
Source: Sci Rep. 2023 Jun 21;13:10105. doi: 10.1038/s41598-023-37241-7 (PMC10284825; doi:10.1038/s41598-023-37241-7)
Supplement: Supplementary file 4 — Supplementary Table S3. [file 41598_2023_37241_MOESM4_ESM.docx]

Table S3 Relative abundance of vaginal microbiota at genus level

| Taxonomy | pre-Control | post-Control | pre-*L.del* intervention | post-*L.del* intervention | pre-Control  vs  post-Control | pre-*L.del* intervention  vs  post-*L.del* intervention | |
| --- | --- | --- | --- | --- | --- | --- | --- |
| Genus (relative %) |  |  |  |  | *P* | *P* | |
| *Lactobacillus* | 0.12±0.04 | 0.08±0.04 | 0.13±0.06 | 0.19±0.06 | 0.1644 | | 0.4996 |
| *Sneathia* | 0.06±0.04 | 0.00±0.00 | 0.02±0.02 | 0.05±0.04 | 0.0158 | | 0.9851 |
| *Escherichia-Shigella* | 0.02±0.01 | 0.04±0.03 | 0.01±0.00 | 0.07±0.04 | 0.8896 | | 0.0236 |
| *Peptoniphilus* | 0.01±0.00 | 0.00±0.00 | 0.05±0.03 | 0.03±0.02 | 0.2884 | | 0.0218 |
| *Gardnerella* | 0.10±0.03 | 0.06±0.03 | 0.12±0.05 | 0.16±0.06 | 0.0493 | | 0.4248 |
| *Ralstonia* | 0.05±0.01 | 0.07±0.01 | 0.01±0.00 | 0.09±0.04 | 0.0120 | | 0.0232 |
| *Streptococcus* | 0.02±0.02 | 0.01±0.00 | 0.13±0.04 | 0.03±0.02 | 0.2102 | | 0.0402 |
| *Finegoldia* | 0.03±0.03 | 0.00±0.00 | 0.00±0.00 | 0.00±0.00 | 0.4619 | | 0.0019 |
| *Brevundimonas* | 0.30±0.04 | 0.41±0.04 | 0.06±0.02 | 0.06±0.02 | 0.0190 | | 0.7541 |
| *Atopobium* | 0.03±0.02 | 0.01±0.00 | 0.03±0.01 | 0.01±0.01 | 0.0078 | | 0.0001 |
| *Prevotella* | 0.04±0.01 | 0.01±0.01 | 0.07±0.02 | 0.01±0.01 | 0.0044 | | 0.0001 |
| *ANPRhizobium* | 0.11±0.02 | 0.14±0.01 | 0.06±0.02 | 0.06±0.02 | 0.1651 | | 0.9881 |
| *Ureaplasma* | 0.01±0.01 | 0.03±0.02 | 0.00±0.00 | 0.00±0.00 | 0.5597 | | 0.7695 |
| *Alloscardovia* | 0.00±0.00 | 0.00±0.00 | 0.02±0.01 | 0.00±0.00 | 0.0280 | | 0.0005 |
| *Anaerococcus* | 0.01±0.00 | 0.01±0.00 | 0.02±0.01 | 0.01±0.01 | 0.2330 | | 0.1256 |
| *Porphyromonas* | 0.00±0.00 | 0.00±0.00 | 0.01±0.01 | 0.00±0.00 | 0.2624 | | 0.3696 |
| *Veillonella* | 0.00±0.00 | 0.00±0.00 | 0.00±0.00 | 0.01±0.01 | 0.6014 | | 0.2964 |
| *Dialister* | 0.01±0.00 | 0.00±0.00 | 0.02±0.01 | 0.00±0.00 | 0.0005 | | 0.0001 |
| *unidentified_Muribaculaceae* | 0.01±0.01 | 0.00±0.00 | 0.00±0.00 | 0.00±0.00 | 0.0836 | | 0.3438 |
| *Peptostreptococcus* | 0.01±0.00 | 0.00±0.00 | 0.00±0.00 | 0.00±0.00 | 0.1792 | | 0.0505 |

Values are presented as means + SEM, Wilcoxon test was used for statistical analysis.
